# Effects of L-Histidine and Sodium Acetate on β-Casein Expression in Nutrient-Restricted Bovine Mammary Epithelial Cells

**DOI:** 10.3390/ani11051444

**Published:** 2021-05-18

**Authors:** Jungeun Kim, Hong-Gu Lee

**Affiliations:** 1 Department of Animal Science and Technology, Sanghuh College of Life Sciences, Konkuk University, Seoul 05029, Korea; sumzzzing@gmail.com; 2 Team of Educational Program for Specialists in Global Animal Science, Brain Korea 21 Plus Project, Konkuk University, Seoul 05029, Korea

**Keywords:** L-histidine, sodium acetate, nutrient restriction, β-casein, bovine mammary epithelial cells

## Abstract

**Simple Summary:**

Nutrient restriction is known to decrease the milk production and milk quality of dairy cows. However, providing cows with abundant nutrients also has a disadvantage because it will increase feed costs. Under such a situation, the use of feed additives can be a good strategy to reduce the feed cost. The objective of this study was to investigate the effects of histidine and sodium acetate on β-casein expression in nutrient-restricted bovine mammary epithelial cells. The results indicate that histidine has the potential to increase the β-casein levels in bovine mammary cells when the nutrient is restricted, suggesting that histidine is a potential feed additive for cows in a nutrient-insufficient environment.

**Abstract:**

Nutrient restriction is a challenging condition for the mammary glands of dairy cows. In this condition, supplementing amino acids and energy sources might be a good strategy to improve the concentration of one of the most important caseins in bovine milk. Therefore, the objective of this study was to investigate the effects of L-histidine (His) and sodium acetate (Ace) in a nutrient-restricted (NR) immortalized bovine mammary epithelial cell line (MAC-T cells). The treatments for the MAC-T cells are as follows: experiment (1) 0–5% diluted basal medium; experiment (2) supplementation of 0–9.6 mM of His or Ace in NR or normal conditions; experiment (3) supplementation of 0–9.6 mM of Ace plus 0.15 mM of His in NR or normal conditions. The 1% diluted medium showed no significant effect on the cell viability with the basal medium; thus, it was selected as the NR condition. The relative expression of β-casein was significantly increased in the NR condition with the inclusion of 0.15 mM His alone or with Ace compared to that in control. The supplementation of Ace increased the β-casein level under normal conditions. However, it did not change the expression of β-casein under the NR condition. The results suggest that His has the potential to increase the β-casein expression under the NR condition.

## 1. Introduction

Lactating cows require significant amounts of nutrients and energy to produce milk for their offspring; thus, nutrient restriction (NR) is a challenging condition for dairy cows. Bovine mammary glands can produce milk proteins. Amino acids (AAs), energy sources, and hormones are essential components for milk protein synthesis [1]. AAs, as components of proteins, have biological roles in animal bodies. Many studies have reported that treatment with AAs can stimulate protein synthesis in mammary epithelial cells in vitro [2,3,4]. Protein synthesis mediated by nutrients is related to the mammalian target of rapamycin (mTOR) pathway [1,5,6]. The mTOR is a central protein complex of the protein synthesis cascade, and is activated by several factors. Once activated, mTOR can phosphorylate ribosomal protein S6 kinase 1 (S6K1) and ribosomal protein S6 (RPS6). Casein gene expression and protein synthesis are then achieved through the translation initiation and elongation process [7].

The supplementation of AA maintained or increased milk production when the cows were fed reduced dietary crude protein [8]. However, knowledge about the effect of single AA on nutrient-restricted (NR) mammary cell levels is limited. Histidine (His), one of essential AAs for dairy cows, maintained the milk protein levels when the cows were fed a metabolizable protein-deficient diet [9,10]. Furthermore, His has been reported to have a stimulatory effect on the mTOR pathway-related genes in mammary epithelial cells [4,11]. Protein synthesis is a highly energy-demanding process. Acetate (Ace) is a precursor of milk fat and an energy source for ruminants, and its ability to stimulate milk protein synthesis have been reported in mammary epithelial levels [12,13]. Thus, His and Ace might be useful for verifying their effects on β-casein mRNA expression in mammary epithelial cells under NR conditions.

Therefore, the objective of the present study was to evaluate the effects of His and Ace on β-casein expression in immortalized bovine mammary epithelial cells under a NR condition in vitro. The hypothesis was that the NR condition would modulate the effects of His and Ace differently from the normal conditions.

## 2. Materials and Methods

### 2.1. Reagents

Dulbecco’s modified Eagle’s medium (DMEM/F12), fetal bovine serum (FBS), penicillin/streptomycin, Pierce™ BCA protein assay kit, and bovine serum albumin (BSA) were obtained from Thermo Scientific (South Logan, UT, USA). Gentamycin, insulin, hydrocortisone, prolactin, L-histidine, and sodium acetate were purchased from Sigma-Aldrich (St. Louis, MO, USA). The cell counting kit-8 (CCK-8) solution was obtained from Dojindo Laboratories (Rockville, MD, USA). Phosphate-buffered saline (PBS) was purchased from Biosesang (Seongnam, Korea). The TRI Reagent was obtained from MRC (Cincinnati, OH, USA). An iScript cDNA synthesis kit was purchased from Bio-Rad (Foster Cty, CA, USA). The AccuPower^®^ 2X GreenStar™ qPCR MasterMix was obtained from Bioneer (Seoul, Korea).

### 2.2. Cell Culture and Experimental Design

**Figure 1:**
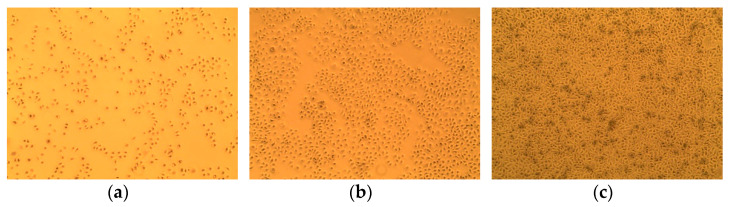
The morphology of the immortalized bovine mammary epithelial cells used in this study (magnification ×10; Olympus Co., Shinjuku, Tokyo, Japan). (**a**) 24 h growth, (**b**) 48 h growth, and (**c**) at the starting time for the treatments of the MAC-T cells in the 6-well plates.

Immortalized bovine mammary epithelial cells (MAC-T cells) were obtained from Dr. Feng-Qi Zhao (University of Vermont, Burlington, VT, USA). The MAC-T cells were incubated as reported previously, with minor modifications [14,15,16]. Briefly, the cells were cultured in 10-cm plates with basal growth medium at 37 °C with 5% CO_2_. The basal growth medium was DMEM/F12 containing 10% FBS, 100 units/mL penicillin/streptomycin, 50 μg/mL gentamycin, 5 μg/mL insulin, and 1 μg/mL hydrocortisone. After this, the cells were transferred to 6-well plates (5 × 10^4^ cells/well) for the treatments. When the cells reached 80% confluence, they were serum-starved overnight and then cultured with a differentiation medium (DIF) (DMEM/F12 containing 100 units/mL penicillin/streptomycin, 50 μg/mL gentamycin, 5 μg/mL insulin, 1 μg/mL hydrocortisone, and 5 μg/mL prolactin) with various treatments for 24 h. The morphology of the MAC-T cells is presented in Figure 1. The treatment media were as follows: experiment (1) basal DIF diluted with distilled water (100%, 99%, 98%, 97%, 96%, and 95%DIF, respectively); experiment (2) 100%DIF or 99%DIF with 0, 0.15, 0.3, 0.6, 1.2, 2.4, 4.8, and 9.6 mM of His or Ace; experiment (3) 100%DIF or 99%DIF plus 0.15 mM of His with 0, 0.15, 0.3, 0.6, 1.2, 2.4, 4.8, and 9.6 mM of Ace. The initial concentrations of the His and Ace in the complete DMEM/F12 were 0.15 mM and 0 mM, respectively. The MAC-T cells were involved in a completely randomized design. In each treatment, an independent well was considered as the experimental unit. All of the experiments were replicated three times on three different days.

### 2.3. Cell Viability

The cell viability was determined using the CCK-8 solution according to the manufacturer’s instructions. Briefly, MAC-T cells were seeded into 96-well plates with 100 μL growth medium (5000 cells/well) and pre-incubated at 37 °C with 5% CO_2_ for 24 h. The spent medium was then replaced for each treatment medium and the cells were incubated for another 24 h. The CCK-8 solution (10 μL/well) was added at 1 h before the end of this 24 h of incubation. The relative cell viability was determined by measuring at the absorbance at 450 nm using a microplate reader (Synergy2, BioTek, Winooski, VT, USA).

### 2.4. Protein Quantification

The cultured supernatants were collected in order to analyze the protein concentration. The samples were centrifuged at 300× *g* for 5 min at 4 °C. The supernatants were then carefully transferred to new tubes in order to measure the protein concentrations using a Pierce™ BCA protein assay kit according to the manufacturer’s instructions by measuring the absorbance of 562 nm using a microplate reader (Synergy2). BSA was used to generate the standard curve.

### 2.5. Gene Expression

After incubation with each treatment medium, MAC-T cells were washed twice with 1 X PBS. After the cells were harvested, the total RNA was extracted using a TRI Reagent. The quantity and quality of the RNA were analyzed using a NanoDrop 1000^®^ Spectrophotometer (Thermo Scientific, Seoul, Korea). RNA with 260/280 ratio of 1.8 to 2.0 and a 260/230 ratio of more than 2.0 were considered acceptable. This RNA was then used for the cDNA synthesis with an iScript cDNA synthesis kit according to the manufacturer’s instructions. The real-time polymerase chain reaction (RT-PCR) was performed using a CFX Connect™ Real-Time System (Bio-Rad, Seoul, Korea) with AccuPower^®^ 2X GreenStar™ qPCR MasterMix in order to determine the target gene expression levels. The PCR conditions were as follows: 95 °C for 3 min, followed by 40 cycles at 95 °C for 10 s, 55–65 °C for 30 s, and 72 °C for 30 s. The primer sequences used in this study are listed in Table 1. β-actin and ubiquitously expressed transcript protein (*UXT*) were used for the normalization of the target genes [17,18]. The target gene expression adjusted by multiple reference genes was conducted using Bio-Rad CFX Manager 3.1 software (Bio-Rad).

### 2.6. Statistical Analysis

The data were analyzed using the GLM procedure of SAS version 9.4 (SAS Institute Inc., Cary, NC, USA). The cell viability, relative protein concentrations, and relative gene expression were analyzed with Tukey’s multiple comparison or *t*-test. The differences between the treatment groups were considered significant at *p* < 0.05.

## 3. Results

### 3.1. Effect of NR on MAC-T Cell Viability

**Figure 2:**
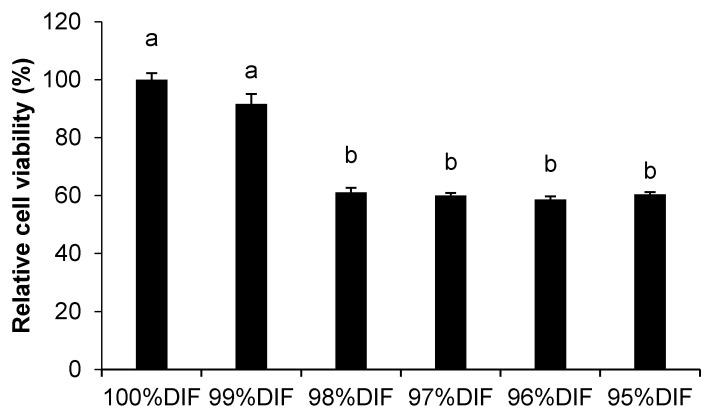
The effect of nutrient-restricted differentiation medium (DIF) on the viability of immortalized bovine mammary epithelial cells (MAC-T). The MAC-T cells were incubated with 0–5% nutrient-restricted DIF for 24 h. The values are presented as the mean ± SEM (*n* = 3). The means without a superscript letter differ (a,b), *p* < 0.05.

The effect of the diluted differentiation medium on the relative MAC-T cell viability is shown in Figure 2. The cell viability decreased with an increase of the dilution rate in the differentiation medium (*p* < 0.001). The treatment group of 99%DIF showed higher relative viability (92%) than the groups treated with further dilutions (≤61%). Thus, 99%DIF was selected as the NR condition for the MAC-T cells.

### 3.2. Individual Effects of His and Ace under the NR Condition

**Figure 3:**
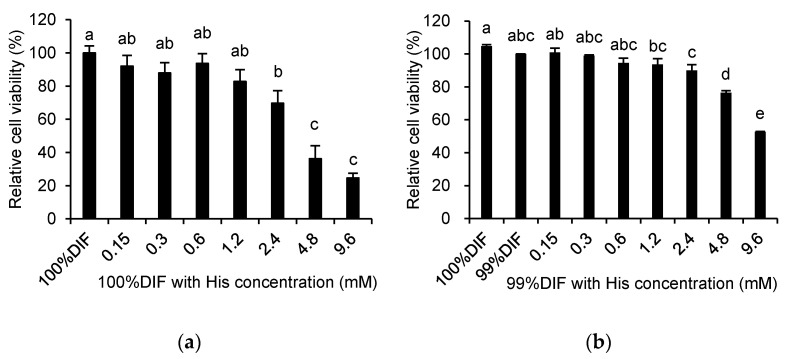

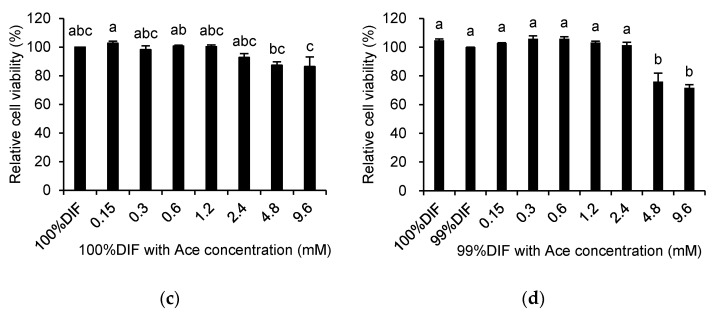
Effects of various concentrations of L-histidine (His) or sodium acetate (Ace) with the unrestricted (100%) or 1% nutrient-restricted (99%) differentiation media (DIF) on the viability of the immortalized bovine mammary epithelial cells (MAC-T). The MAC-T cells were incubated with (**a**) 100%DIF plus His, (**b**) 99%DIF plus His, (**c**) 100%DIF plus Ace, or (**d**) 99%DIF plus Ace for 24 h. The values are presented as the mean ± SEM (*n* = 3). The means without a superscript letter differ (a–e), *p* < 0.05.

The effects of His, Ace, or NR on the relative viability of the MAC-T cells are shown in Figure 3. The relative cell viability was decreased by His in a dose-dependent manner regardless of the NR condition (*p* < 0.0001, Figure 3a,b). The cell viability was also decreased by Ace in a dose-dependent manner under the 100%DIF condition (*p* = 0.004, Figure 3c). Statistical changes of the cell viability were not observed for the groups treated with 0–2.4 mM of Ace and 99%DIF. However, the relative cell viability was decreased in groups treated with 4.8–9.6 mM of Ace and 99%DIF (*p* < 0.001, Figure 3d). For the subsequent analyses, the treatment groups that showed no statistically significant difference in their cell viability compared to the group treated with 100%DIF without any supplementation of His or Ace were selected: 0–1.2 mM of His with 100%DIF, 0–0.6 mM of His with 99%DIF, 0–2.4 mM of Ace with 100%DIF, and 0–2.4 mM of Ace with 99%DIF.

**Figure 4:**
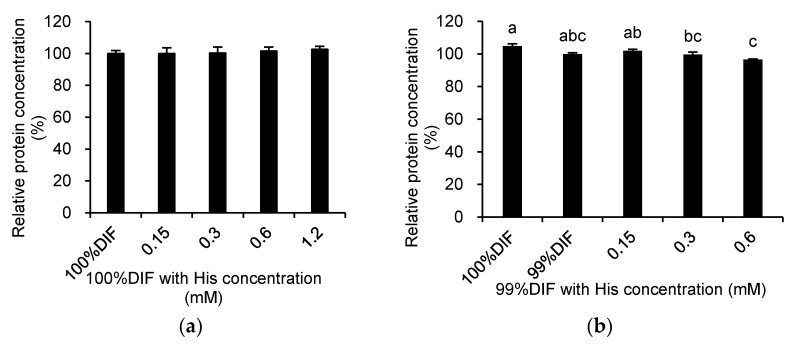

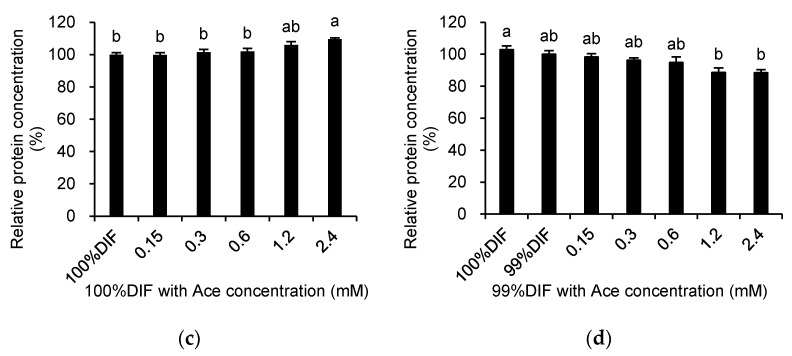
Effects of various concentration of L-histidine (His) or sodium acetate (Ace) with unrestricted (100%) or 1% nutrient-restricted (99%) differentiation media (DIF) on the relative protein concentrations in the culture medium of the immortalized bovine mammary epithelial cells (MAC-T). The MAC-T cells were incubated with (**a**) 100%DIF plus His, (**b**) 99%DIF plus His, (**c**) 100%DIF plus Ace, or (**d**) 99%DIF plus Ace for 24 h. The values are presented as the mean ± SEM (*n* = 3). The means without a superscript letter differ (a–c), *p* < 0.05.

The relative medium protein concentrations in the medium after the treatment of the MAC-T cells with His or Ace are shown in Figure 4. The treatment with His did not affect the protein concentration under the 100%DIF condition (*p* > 0.10, Figure 4a). The treatment with His under the NR condition showed similar patterns of cell viability (*p* = 0.003, Figure 4b). The addition of Ace into 100%DIF dose-dependently increased the relative concentration of the secreted proteins (*p* = 0.005, Figure 4c). Conversely, the Ace supplementation in 99%DIF dose-dependently decreased the relative concentration of the secreted proteins (*p* = 0.005, Figure 4d). Hence, the relative β-casein mRNA levels in the groups treated with 99%DIF and His or 100%DIF with Ace were analyzed, as these groups showed statistically significant differences in cell viability.

**Figure 5:**
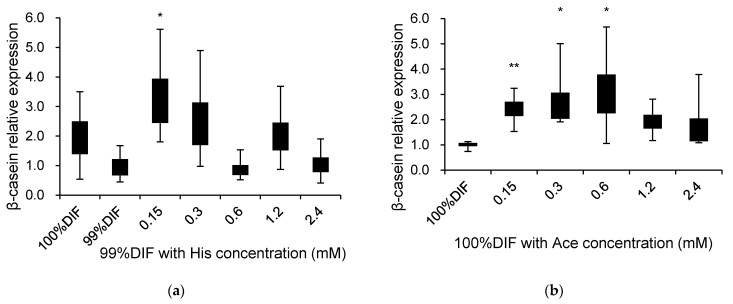
Effects of various concentrations of L-histidine (His) or sodium acetate (Ace) with unrestricted (100%) or 1% nutrient-restricted (99%) differentiation media (DIF) on the β-casein mRNA expression in immortalized bovine mammary epithelial cells (MAC-T). The MAC-T cells were incubated with (**a**) 99%DIF plus His, or (**b**) 100%DIF plus Ace for 24 h. The values are presented as the median and interquartile ranges (*n* = 3). Significant difference compared to the control was presented as * *p* < 0.05, ** *p* < 0.01.

The effects of His, Ace, or NR on the relative β-casein mRNA expression are presented in Figure 5. The multiple comparisons were not observed between the treatment groups. For the His-treated groups, 0.15 mM with 99%DIF increased the relative expression of β-casein 2.2-fold compared to 99%DIF without His supplementation (*p* = 0.014, Figure 5a). The delta Ct values of each treatment in Figure 5a were as follows: 100%DIF = 9.82, 99%DIF = 10.77, 99%DIF with 0.15 mM of His = 9.07, 99%DIF with 0.3 mM of His = 9.42, 99%DIF with 0.6 mM of His = 10.96, 99%DIF with 1.2 mM of His = 9.73, and 99%DIF with 2.4 mM of His = 10.76. The stimulatory effect of Ace on the β-casein mRNA level was observed under the 100%DIF condition (*p* < 0.05, Figure 5b). When 0.15, 0.3, or 0.6 mM of Ace was supplemented in the 100%DIF condition, the β-casein mRNA level was increased 1.4-fold, 1.7-fold, and 2.1-fold, respectively, compared to the 100%DIF condition. The delta Ct values of each treatment in Figure 5b were as follows: 100%DIF = 13.45, 100%DIF with 0.15 mM of Ace = 12.19, 100%DIF with 0.3 mM of Ace = 12.08, 100%DIF with 0.6 mM of Ace = 11.95, 100%DIF with 1.2 mM of Ace = 12.53, and 100%DIF with 2.4 mM of Ace = 12.79. Therefore, based on 0.15 mM of His, which revealed an increase in the β-casein mRNA level, an additional experiment was conducted in order to confirm the combination effect of His and Ace.

### 3.3. Combination Effects of His and Ace in the NR Condition

**Figure 6:**
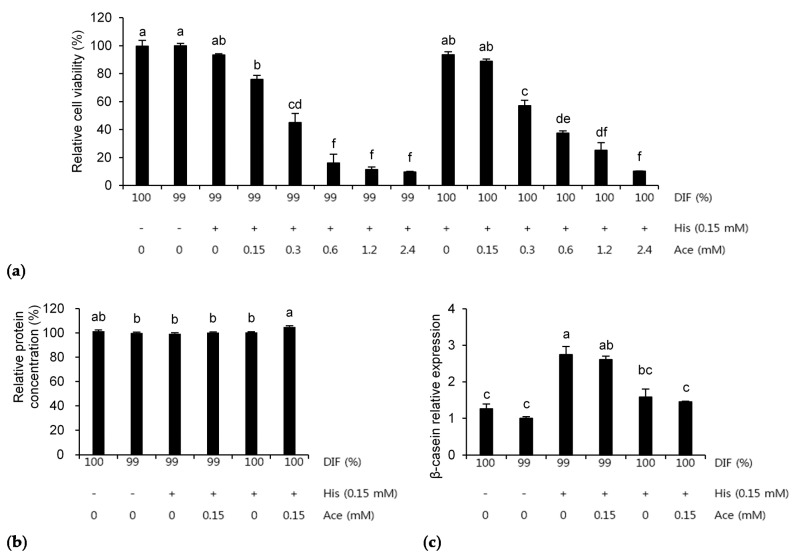
Effects of 0.15 mM of L-histidine (His) plus various concentrations of sodium acetate (Ace) with unrestricted (100%) or 1% nutrient-restricted (99%) differentiation media (DIF) on (**a**) the relative cell viability, (**b**) the relative protein concentration in the cultured medium, and (**c**) the β-casein mRNA expression in the immortalized bovine mammary epithelial cells (MAC-T). The MAC-T cells were incubated for 24 h after each treatment. The values are presented as the mean ± SEM (*n* = 3). The means without a superscript letter differ (a–f), *p* < 0.05.

The effects of NR or 0.15 mM of His plus various concentrations of Ace on the MAC-T cells are presented in Figure 6. The relative cell viability was decreased by Ace dose-dependently in both the NR and unrestricted conditions (*p* < 0.001, Figure 6a). Therefore, six treatments were selected for the further analyses. When 0.15 mM of His and Ace was supplemented in the 100%DIF condition, the relative protein concentration in the medium was increased significantly compared to the other groups, except for the 100%DIF group (*p* = 0.002, Figure 6b). However, the mRNA expression levels of β-casein were increased 1.7-fold and 1.6-fold in the treatment groups of His and His plus Ace with 99%DIF (*p* = 0.003, Figure 6c). The delta Ct values of each treatment in Figure 6c were as follows: 100%DIF = 13.45, 99%DIF = 13.68, 99%DIF with 0.15 mM of His = 12.16, 99%DIF with 0.15 mM of His and Ace = 12.24, 100%DIF with 0.15 mM of His = 13.38, and 100%DIF with 0.15 mM of His and Ace = 13.40.

The relative expression levels of the genes involved in protein synthesis and nutrient transportation after treatment with His, Ace, or NR are presented in Table 2. The treatments did not affect the mRNA expression levels of sodium-dependent neutral amino acid transporter type 2 (*ASCT2*), *mTOR*, or *RPS6* (*p* > 0.10). However, the mRNA level of large neutral amino acids transporter small subunit 1 (*LAT1*) in the group treated with 0.15 mM His plus 0.15 mM Ace in 100%DIF was increased compared to that in other groups (*p* = 0.004). The expression levels of *LAT1* and glucose transporter 1 (*GLUT1*) were increased by 39% and 45% in the 100%DIF condition compared to those in the 99%DIF condition, respectively (*p* < 0.01). A significant combination effect of His and Ace on the expression levels of *LAT1* and *S6K1* compared to the 100%DIF condition was observed when the nutrient was not restricted (*p* < 0.01).

## 4. Discussion

When Holstein × Normande crossbreed dairy cows were in NR, the DNA concentration of their mammary glands was significantly decreased compared to unrestricted cows [19]. Moreover, the mammary glands of the NR cows showed more apoptotic cells and increased mRNA levels of apoptosis-related genes in the same study. Viable mammary epithelial cells exfoliated from the mammary glands tended to increase in the milk of NR Holstein cows compared to those in the control cows [20]. The aforementioned studies reported the decreased viability of mammary cells when the dairy cows were in the NR condition. These were distinguished in our study (in vivo vs. in vitro); however, similar results were observed with our cell viability results in experiment 1.

Studies on the AA supply in mammary epithelial cells have reported increased cell viability through AA; however, a high dose of AA inversely decreased the viability [4,21,22]. Similarly to the results of other AAs, the supplementation of His in an AA-devoid cell medium increased the relative growth rate of Chinese mammary epithelial (CMEC-H) cells compared to the control, and an excess dose of His decreased the cell growth rate [4]. This partially agreed with the results of our study. The lack of an increase in the relative cell viability was probably due to the use of an AA-adequate medium in this study. His was supplemented to the AA-containing medium; a high concentration of AA in the medium may have had adverse effects on the cell viability. The supplementation of 4–8 mM Ace increased the relative growth rate in pbMECs [23]. The cell viability was not affected by supplementation with 0.25–5 mM Ace in primary bovine mammary epithelial cells (pbMECs) isolated from mammary tissues [24]. This is consistent with our results using MAC-T cells. These results suggest that a high dose of Ace has cell toxicity, and that MAC-T cells can show similar responses to pbMECs.

The relative expression of β-casein in CMEC-H cells was significantly increased when 0.15 mM of His was supplemented in an AA-devoid medium [4,11]. Comparing these results and our previous study, the inclusion of 0.15 mM His has the potential to stimulate β-casein synthesis, although in an AA-existent NR condition. Thus, 0.15 mM His was selected for the verification of its combination effect with Ace. The supplementation of Ace presented an increase of the β-casein mRNA level in pbMECs cultured with DMEM/F12 [13]. This is consistent with our results when 0.15–0.6 mM Ace was supplemented to the MAC-T cells. The decrease of the protein concentration in the NR groups might be because the cells used supplemented Ace as an energy source. Therefore, as shown in Figure 5b, the present study suggests that Ace can stimulate β-casein expression when nutrients are abundant.

The relative cell viability was lowered by Ace in a dose-dependent manner. Ace did not show a great effect on the MAC-T cell viability in experiment 2; however, when Ace was mixed with 0.15 mM His, the cell viability was dose-dependently decreased. Thus, the decrease of the MAC-T cell viability was considered to be due to the effect of His. It has been reported that Ace has a synergistic effect on secretory proteins when it is supplemented with AA. When Ace and methionine were used together to treat MAC-T cells with an AA-adequate medium, the quantity of the secreted proteins increased compared to that in the group treated with methionine alone [25]. In our results, a synergistic effect on the relative protein concentration in the cultured medium was observed when both 0.15 mM of His and Ace were used for the treatment under normal conditions. The results suggest that a supply of Ace with His has stimulatory effects on protein synthesis. Some synergistic effects on β-casein expression were reported when Ace was treated with leucine [13] or methionine [25] in nutrient-abundant conditions. Although the protein concentration in the cultured medium was increased after the treatment with a combination of Ace and His, such a combination did not show a synergistic effect on β-casein mRNA expression under the 100%DIF condition. According to Gao et al. (2015), the supplementation of 0.15 mM His to the CMEC-H cells mostly increased the expression of β-casein and κ-casein (compared to a further dose of His); however, the inclusion of 0.15 mM His did not increase the αs1-casein expression. Thus, the increased protein concentration was maybe due to other caseins and proteins, rather than β-casein. Further research on various milk proteins is needed in order to determine the interaction between His and Ace.

ASCT2 is an AA transporter that absorbs branched-chain AAs, threonine, and some nonessential AAs; LAT1 transports essential AAs except Lys, Arg, and Thr [5]. In our previous study, the expression of *ASCT2* was increased when 0.6 mM of L-valine (Val) was supplemneted to MAC-T cells, whereas Val did not affect the *LAT1* expression [14]. The supplementation of L-phenylalanine (Phe) did not affect the expression of *ASCT2* or *LAT1* either. Dai et al. (2019) reported that Met supplementation increased *ASCT2* expression by through the seryl-tRNA synthetase-mediated β-casein synthetic pathway. Thus, the literature reported that the effects of various AAs on cells are different, and in the present study, it was found that His had no effect on the expression of AA transporters. *GLUT1* is used as a major glucose tansporter in various cells and tissues, including the bovine mammary gland that absorbs basal glucose [26,27]. The expression of *GLUT1* increased when glucose was supplemented to pbMECs [28]. When dairy cows were subjected to NR, the *GLUT1* expression decreased in pbMECs from bovine milk from NR cows [29]. The reports suggest that the *GLUT1* expression in MECs is influenced by their energy state. This is consistent with our finding that *GLUT1* exression was increased in 100%DIF compared to 99%DIF. Zhao et al. (2019) reported an increase of cellular ATP concents by the supplementation of Ace. Ace was not affected by the *GLUT1* levels in this study; however, it may be a potential energy source in mammary epithelial levels.

Protein synthesis is regulated by the mTOR complex and its downstream pathway, including S6K1 and RPS6 [1]. mTOR signaling is a highly energy-requiring process, and is regulated by AA and energy sources [1,6]. Thus, various results have been reported for the regulation of mTOR pathway-related genes by AAs [30,31] or ratios of AAs [32,33,34]. An increase of *mTOR* was observed when 0.15 mM His was supplemented to CMEC-H cells cultured in an AA-devoid medium [4,11]. However, the changes of the *mTOR* expression between the groups were not confirmed in the present study. This might be explained by the different medium conditions: AA-devoid medium in previous studies [4,11] and DMEM/F12 in the present study. The supplementation of 4–12 mM Ace increased the *mTOR* mRNA levels in pbMECs [13]. In the present study, 0.15 mM Ace might have been a dose that was too low to increase the *mTOR* expression. Previous reports have shown various responses of *S6K1* expression with His supplementation: decrease [35], increase [4,11], and no change [31]. Although the results are inconsistent, the increase of *S6K1* in the present study might be related to the use of an AA-containing medium. *RPS6* is a downstream gene of *S6K1*, and is stimulated by *S6K1*. However, the gene network is highly comxplex; thus, increased *S6K1* expression does not always stimulate *RPS6* expression. The expression of total *S6K1* was increased by a differing Lys:Met ratio (2.5:1) compared to the control (Lys:Met = 2.9:1) when the MAC-T cells were in the thermal neutral condition (37 °C); however, the total *RPS6* was not changed [33]. The phosphorylation states of *mTOR* and *S6K1* were not changed by differing Lys:Thr, Lys:His, and Lys:Val ratios (2.1:1, 3.05:1, and 1.62:1, respectively) compared to the control; however, the *RPS6* phosphorylation state was increased by these treatments [36].

His supplementation did not affect the mRNA levels of mTOR pathway-related genes. However, it increased the β-casein expression level in the NR groups. Thus, His might stimulate the expression of β-casein in the NR condition through other pathways. Further studies are needed in order to verify the effects of His when nutrients are limited.

## 5. Conclusions

Overall, 99%DIF was selected as the NR condition for MAC-T cells. The supplementation of His dose-dependently decreased the cell viability regardless of the NR condition. The β-casein mRNA level clearly increased in the 0.15 mM His-supplemented NR group. The group with Ace supplementation showed higher MAC-T cell viability than the His-treated groups. When Ace was used for the treatment under the normal medium condition, the expression of β-casein was increased. In combination with Ace, His dominantly affected the MAC-T cell viability. The relative protein concentration increased when 0.15 mM of His and Ace were used for the treatment under normal conditions; however, the β-casein expression was increased by His alone, or when both His and Ace were supplemented in the NR condition. Overall, these results suggest that His has a potential to increase the expression of β-casein in MAC-T cells under NR.

## Figures and Tables

**Table 1:** List of primer sequences used for the real-time polymerase chain reaction (RT-PCR) analysis.

Gene ^1^	Accession No.	Type ^2^	Sequence (5′-3′)
*ASCT2*	NM_174601.2	F	TGCCGCTGATGATGAAGTGT
		R	AGTCCACGGCCAAGATCAAG
*GLUT1*	NM_174602.2	F	TCGCTTCATCATCGGTGTGT
		R	GCTTCTTCAGCACGCTCTTG
*LAT1*	AF174615	F	TACTTCCTTGGGGTCTGGTG
		R	GTATCTGCGGACATCCACCT
*mTOR*	XM_002694043.6	F	ATGCTGTCCCTGGTCCTTAT
		R	GGGTCAGAGAGTGGCCTTCA
*RPS6*	NM_001015548.2	F	TGAAGCAGGGTGTCTTGACC
		R	TCCAGTCCTCCTTGGTCTGT
*S6K1*	NM_205816.1	F	GGACATGGCAGGGGTGTTT
		R	GGTATTTGCTCCTGTTACTT
*UXT*	NM_001037471.2	F	GCGCTACGAGGCTTTCATCT
		R	CCAAGGGCCACATAGATCCG
β-actin	NM_173979.3	F	GCATGGAATCCTGCGGC
		R	GTAGAGGTCCTTGCGGATGT
β-casein	XM_015471671.2	F	GAGCCTGACTCTCACTGATGTTGAA
		R	GACAGCACGGACTGAGGAGGAA

^1^ *ASCT2* = sodium-dependent neutral amino acid transporter type 2; *GLUT1* = glucose transporter 1; *LAT1* = large neutral amino acids transporter small subunit 1; *mTOR*; mammalian target of rapamycin; *RPS6* = ribosomal protein S6; *S6K1* = ribosomal protein S6 kinase 1; *UXT* = ubiquitously expressed transcript protein. ^2^ F = forward; R = reverse.

**Table 2:** Effects of L-histidine and sodium acetate on the expression levels of nutrient transporters and the mammalian target of rapamycin pathway genes in nutrient-restricted or unrestricted immortalized bovine mammary epithelial cells.

	Treatment ^1,2^							
			99%DIF + His		100%DIF + His			
Gene ^3^	99%DIF	100%DIF	Ace −	Ace +	Ace −	Ace +	SEM ^4^	*p*-Value
*ASCT2*	1.00	1.09	0.79	0.86	1.11	1.32	0.11	0.314
*LAT1*	1.00 ^b^	1.39 ^b^*	0.86 ^b^	0.82 ^b^	1.02 ^b^	1.84 ^a#^	0.13	0.004
*GLUT1*	1.00	1.45 *	0.85	1.08	0.99	1.38	0.13	0.068
*mTOR*	1.00	1.05	0.87	0.93	0.97	1.16	0.06	0.582
*S6K1*	1.00	0.99	0.98	1.02	0.96	1.38 ^#^	0.05	0.221
*RPS6*	1.00	0.97	1.06	0.95	1.00	1.00	0.02	0.590

^1^ 99%DIF = 1% diluted differentiation medium; 100%DIF = differentiation medium; His = 0.15 mM of L-histidine; Ace = 0.15 mM of sodium acetate. ^2^ Least squared means; *n* = 3 (n is the number of observations used in the statistical analysis). ^3^ *ASCT2* = sodium-dependent neutral amino acid transporter type 2; *GLUT1* = glucose transporter 1; *LAT1* = large neutral amino acids transporter small subunit 1; *mTOR*; mammalian target of rapamycin; *RPS6* = ribosomal protein S6; S6K1 = ribosomal protein S6 kinase 1; *UXT* = ubiquitously expressed transcript protein. ^4^ SEM = standard error of the mean. ^a–b^ Within a row, the means without a superscript letter differ (*p* < 0.05). * A significant difference compared to 99%DIF (*p* < 0.05). ^#^ A significant difference compared to 100%DIF (*p* < 0.05).

## Data Availability

The data presented in this study are available on request from the corresponding author.
